# Reverse vaccinology approach towards the in-silico multiepitope vaccine development against SARS-CoV-2

**DOI:** 10.12688/f1000research.36371.1

**Published:** 2021-01-23

**Authors:** Vipul Kumar, Sudhakar Kancharla, Prachetha Kolli, Manoj Jena

**Affiliations:** 1 Department of Biotechnology, School of Bioengineering and Biosciences, Lovely Professional University, Phagwara, Punjab, 144411, India; 2 Devansh Lab Werks, Homewood, AL, 35209, USA; 3 Microgen Health Inc, Chantilly, VA, 20151, USA

**Keywords:** SARS-CoV-2, COVID-19, Immunoinformatics, multiepitope, docking, simulations.

## Abstract

**Background**: The novel severe acute respiratory syndrome related corona virus-2 (SARS-CoV-2) belongs to the “Coronaviridae” family and order “Nidovirales”, which has caused the pandemic coronavirus disease 2019 (COVID-19). SARS-CoV-2 has been spread in more than a 100 countries, and more than a million have lost their lives. Vaccination and immunization could be an effective strategy to combat fatal COVID-19.

**Methods**: For identification of effective vaccine candidate against COVID-19, various immunoinformatics online tools and softwares were used to predict epitopes. Cytotoxic T cell epitopes, helper T cell epitopes, and B cell epitopes from three structural polyproteins (Spike, Membrane, and Nucleocapsid (SMN) based on the binding affinity towards MHC, antigenicity, non-allergenicity, and non-toxicity) were identified for vaccine development. The multiepitope based vaccine was constructed linking two additional adjuvants human beta-defensin-3 and human beta-defensin-2 at N and C terminal, respectively.

**Results**: The constructed vaccine sequence was found to be a good antigen and non-allergen for the human body. The constructed vaccine was docked with the TLR-3 receptor.  The docked complex was further taken for molecular dynamics simulations and RMSD was calculated, which showed stable binding of the complex. The codon adaptation index (CAI) of 0.92 and GC content of 55.5% for *E. coli* (K12 strain) suggested efficient expression of the predicted vaccine.

**Conclusion**: The current study can be helpful in the reduction of time and cost for further experimental validations and could give a valuable contribution against this pandemic.

## Introduction

Coronaviruses (CoVs) belong to the family of coronaviridae in the order Nidovirales, and have single-strand positive-sense RNA ^1^. The size of the RNA of coronavirus is the largest among the viruses (~30 kb) ^2^. They have glycoprotein projections on the envelope, which gives the corona appearance. CoVs are pathogens mainly involved in respiratory and gastrointestinal diseases in a wide range of animals and humans ^1^^,^^2^. CoVs are divided into four sub-categories, namely alpha, beta, gamma, and delta, out of which alpha and beta coronavirus are known to infect humans ^1^^,^^3^. From alpha and beta, four strains are responsible for the common cold, and two strains were found to be responsible for severe acute respiratory syndrome (SARS-CoV) and Middle East respiratory syndrome CoV (MERS-CoV) ^4^^,^^5^. Recently in December 2019, a novel coronavirus SARS-CoV-2 was detected from patients of novel coronavirus disease 2019 (COVID-19) in the Wuhan, China ^6- 8^. The symptoms of COVID-19 infection include headache, fever, pneumonia, and asthenia ^9^^,^^10^. A severe and fatal outbreak of this virus has taken many lives and has created enormous economic loss worldwide. The treatment and prevention from this infection is the need of the hour.

COVID-19 has given a serious and tough challenge to biomedical research scientists and researchers all around the world. Much research is looking at repurposing antiviral drugs, and developing new drugs and vaccines against the SARS-CoV-2 ^11- 15^. Here, an attempt has been given to construct an in-silico vaccine, which can be further validated through experimental assays and could play a major role in the management of this pandemic. In this study, three structural proteins form SARS-CoV-2, based on the antigenicity has been selected for the construction of the vaccine. The first structural protein is Spike (S) glycoprotein, which has been reported to be a crucial surface protein of SARS-CoV-2, which facilitates the entry of the virus inside the host cell. It has been reported that for the entry of the SARS-CoV-2, S protein first binds with Angiotensin-Converting Enzyme-2 (ACE-2) receptor, and then is primed by the host serine protease (TMPRSS2) ^16^^,^^17^. This priming of the S protein lets it to fuse into the host cell membrane and entry inside the cell. The second crucial structural protein, which induces a strong immune response, is Membrane (M) glycoprotein. It plays a crucial role in virus morphogenesis and assembly by interacting with several other viral proteins ^18^. The third structural protein chosen for vaccine construct is Nucleocapsid (N) phosphoprotein; it links the viral genome to the envelope. It consists of two domains, N terminal and C terminal, and both can bind to RNA. It has been reported that the C terminal domain facilitates the physical interaction of the RNA genome and envelope ^19^^,^^20^. All these three structural proteins are predicted to be good antigens and could induce the immune response.

In this pandemic situation, an immunoinformatics approach could be a fast, scientifically sound, and reliable option for quicker vaccine development. These three proteins chosen for the present study were predicted to be good antigens, which gives the opportunity to predict B and T cell epitopes. When naive B cells interact with the antigenic B cell epitopes via its transmembrane bound antibody, they differentiate into two types of cells plasma and memory cells ^21^^,^^22^. Plasma cells lack receptors, but they produce a large number of antibodies against the antigen. Memory cells express membrane-bound antibody molecules, but they are functionally inactive unless they encounter the same antigen again ^23- 25^. Furthermore, T cell epitopes are recognized by Major Histocompatibility Class (MHC), a glycoprotein present on the variety of the cells, which display the antigen to T cells ^26^^,^^27^. Antigen-presenting MHCs are divided into major two classes, MHC class-I are expressed on nucleated cells while MHC class-II are only expressed by antigen-presenting cells. Class II MHC interacts with T helper cells and activates B cells via cytokines, while Class-I MHC interacts with cytotoxic T cells, which kills virus-infected host cells ^28^^,^^29^. Further to know the ability of a constructed vaccine for inducing innate as well as antigen-specific acquired immunity, the constructed vaccine must dock with Toll-Like Receptor-3 (TLR-3). TLRs are mainly expressed on various leukocytes such as dendritic cells, natural killer cells, and cells of adaptive immunity such as T cells and B cells ^30^^,^^31^. Hence, in this study, an attempt has been made to construct the multiepitope vaccine consists of Helper T cells (HTLs), Cytotoxic T Cells (HTLs), and B cell epitopes, which could interact with TLR-3 and generate the immune response. This constructed multiepitope vaccine may induce both humoral as well as cell-mediated immune responses.

## Methods

### Retrieval of structural polyproteins of the SARS-CoV-2

The complete sequence of all three structural polyproteins from SARS-CoV-2 reference sequence ( NC045512.2) were retrieved from NCBI on the basis of their antigenicity. The spike (S) glycoprotein ( YP_009724390.1), Membrane (M) glycoprotein ( YP_009724393.1) and Nucleocapsid (N) phosphoprotein ( YP_009724397.2) were retrieved in FASTA format. These three proteins together are referred to as SMN (Spike, Membrane, and Nucleocapsid) polyprotein in this study.

### Cytotoxic T cell epitopes (CTL) prediction

First, the CTL epitopes for SMN polyproteins were predicted using Netctl 1.2 server ^32^. Prediction of the epitopes depends on three major attributes: (1) binding affinity of MHC-1 class; (2) ability of the proteasome cleavage; and (3) TAP transport efficiency. The first two are predicted with the artificial neural network algorithm while third one using weight matrix. For the prediction of the epitopes threshold for epitopes, identification was chosen to be 0.75, weight on C terminal cleavage was set on 0.15, while the weight on TAP transport efficiency was set on 0.05. The predicted epitopes were ranked according to the combined score.

### Helper T cell (HTL) epitope prediction

For the prediction of HTL epitopes, the IEDB MHC II server was used ^33^. The species/locus was selected as Human/HLA-DR, and a 7-allele HLA reference set was selected for the prediction. Further, 15 mer length of the epitopes were retrieved and ranked according to the percentile. The percentile rank is given after comparing the peptides score with five million 15 mers from the SWISSPROT database. The higher percentile value means a lower binding affinity of MHC-II. For further refinements of the HTL epitopes, these selected HTL epitopes were subjected to investigate whether they can induce IFN gamma immune response using the IFN epitope server ^34^. For IFN gamma inducing epitopes selection, the Motif/SVM hybrid approach was chosen, and the model was set to be IFN gamma versus non-IFN gamma. Finally, the epitopes whose results were positive for the IFN gamma response were chosen for the in-silico vaccine development.

### B cell epitope prediction

B cell epitopes were predicted using the ABCpred server ^35^. This server predicts B cell epitopes using recurrent neural network algorithm. For the identification of the epitopes, the threshold was set on 0.51, while the window length for the prediction was chosen to be 16, keeping overlapping filter on. Top predicted epitopes having scored more than 0.9 was only chosen for the development of the candidate vaccine. Further, after the construction of the vaccine, linear as well as discontinuous conformational B cell epitopes were identified in the vaccine construct using ElliPro, an online server ^36^. Elipro predicts the antibody epitopes taking protein antigen tertiary structure as input.

### Antigenicity, allergenicity, and toxicity prediction

The important attributes such as the antigenicity, allergenicity and toxicity were predicted for all the predicted epitopes individually as well as after construction of the vaccine. First of all, the antigenicity was investigated using the VaxiJen 2.0 server ^37^, and only probable antigen epitopes were chosen for the construction of the vaccine. Further, the allergenicity was predicted using the AlgPred server ^38^ and only non-allergenic epitopes were selected. Finally, all the epitopes were investigated for toxicity using the ToxinPred server ^39^ and non-toxic epitopes were selected. All the predicted epitopes had to cross all these barriers. The overall construct of the vaccine was also tested for these attributes.

### Construction of multiepitope vaccine sequence

The vaccine sequence was constructed using the best identified CTL, HTL, and B cell epitopes. For the construction of the sequence at the N terminal and C terminal, an adjuvant was added using EAAAK linkers. While HTL epitopes were linked using GPGPG, linkers and CTL epitopes were linked using AAY linkers. In the C terminal, HHHHHH was added for the easy purification of the vaccine.

### Physiochemical properties of the vaccine sequence

The physicochemical properties such as molecular weight, PI, half-life, aliphatic index, and hydropathicity were predicted using online tool ProtParam ^40^.

### Secondary structure of the vaccine sequence

Protein secondary structure prediction gives further opportunity to predict the tertiary structure as well as gives information about the activity and function of the protein. The secondary structure of the final multiepitope vaccine sequence was predicted by the free online web tool CFSSP ^41^.

### Tertiary structure prediction

The tertiary structure of the constructed vaccine was predicted using the Rosetta web tool ^42^. Rosetta tool applies a deep neural network algorithm to predict the inter-residue distances as well as orientations. Then these orientations are converted to smooth inter-residue constraints followed by gradient descent energy minimization. Further, the coarse-grained models are generated, and full atom refinement is done. The validation of the model has been done through Ramachandran plot analysis using VADAR web tool ^43^. Further, the modelled structure was validated through the ProSA web tool ^44^, which gives the quality Z score of the modelled protein based on the already known similar size of the proteins crystal structures.

### Minimization and equilibration of the predicted structure

To achieve a more stable structure, the predicted structure was further taken for molecular dynamics (MD) simulations using Gromacs software ^45^. The structure was minimized using the steepest descent algorithm with 50000 steps, followed by NVT and NPT equilibration for 100 picoseconds, followed by MD simulations of 500 picoseconds. The last frame of the MD trajectory was taken for further analysis.

### Molecular docking of constructed vaccine with TLR-3 receptor

For molecular docking, the last frame from the MD simulations of the constructed vaccine was taken, and the TLR-3 structure was retrieved from Protein Data Bank (PDB; ID 1ZIW). The downloaded structure was prepared and processed for docking using dock prep tool UCSF Chimera software. For the docking, the vaccine construct and TLR-3 was uploaded to patchDock server ^46^. Further, for refinement of the rigid body molecular docking solutions, FireDock server was used ^47^. It gave the best 10 docked confirmation based on global energy and Van der Waal’s interactions.

### Reverse translation and codon optimization

Finally, for expressing the constructed multiepitope, the vaccine needs to be expressed in the suitable vector inside the prokaryotic system. Hence, reverse translation and codon optimization were analysed using the Java codon adaptation web tool ( Jcat) ^48^. The codon optimization was performed for *E. coli* strain K12 as a host. Jcat gives the codon adaption index (CAI) and percentage GC content as output. The CAI gives the information of codon usage, generally score between 1 and 0.8, while GC contents should be between 40 % to 70%, values lying outside the given margin is suggested to be inefficient ^49^.

## Results

### Retrieval of the polyproteins and antigenicity

The amino acid sequence of all three (SMN) structural proteins were retrieved from the NCBI database in fasta format. The proteins were investigated for antigenicity by Vaxijen web tool, and it was found that all the three chosen proteins could be good antigens. The default threshold of 0.4 was chosen as the criteria for the antigenicity in the Vaxijen tool. The spike protein showed a score of 0.46; membrane glycoprotein showed a score of 0.51; while nucleocapsid protein showed a score of 0.50. Hence, all three proteins were chosen for further predictions of B cell and T cell epitopes and the construction of the vaccine.

### Prediction of CTL epitopes

CTL epitopes were predicted using Netctl 1.2 server for all the three selected proteins. A total of 38 epitopes was predicted from spike glycoprotein; 10 epitopes were predicted from membrane glycoprotein; while 9 were predicted from nucleocapsid protein. Out of all these predicted CTL epitopes, only 8 were selected for the construction of the vaccine, based on a high binding affinity towards MHC-I, antigenicity, non-allergenicity, and non-toxicity predictions, as shown in **Table 1**.

**Table 1.** List of the final selected CTL epitopes which have fulfilled all the criteria for antigenicity, non-allergenicity, non-toxicity and could bind efficiently to MHC-I.

Peptide sequence	MHC binding affinity	Rescale binding affinity	C -terminal cleavage affinity	Transport efficiency	Prediction score	Polyprotein
WTAGAAAYY	0.6735	2.8596	0.7339	2.8630	3.1128	S protein
WMESEFRVY	0.3902	1.6569	0.7993	2.9290	1.9232	S protein
AGDSGFAAY	0.1341	0.5695	0.9652	2.6730	0.8480	M protein
LVGLMWLSY	0.2694	1.1440	0.7240	2.8970	1.3974	M protein
VATSRTLSY	0.2752	1.1684	0.9679	3.0130	1.4642	M protein
LSPRWYFYY	0.4837	2.0538	0.9746	2.8150	2.3408	N protein
DLSPRWYFY	0.2866	1.2167	0.9760	2.7250	1.4994	N protein
NTASWFTAL	0.1772	0.7523	0.9557	1.1280	0.9521	N protein

### Prediction of HTL epitopes

HTL epitopes were predicted using the IEDB MHC II server for all the three SMN structural proteins. Finally, 4 HTL epitopes were selected on the basis of binding affinity, antigenicity, non-allergenicity, and non-toxicity, as shown in **Table 2**. Four human alleles and position of predicted epitopes are HLA-DRB1*07:01 (166-180), HLA-DRB4*01:01 (298-312), HLA-DRB5*01:01 (232-246), HLA-DRB5*01:01 (345-359).

**Table 2.** List of the final selected HTL epitopes which fulfilled all the criteria for antigenicity, non-allergenicity, non-toxicity and could also induce the IFN gamma immune response.

Allele	Start	End	Peptide sequence	Percentile score	Polyprotein
HLA-DRB5*01:01	232	246	GINITRFQTLLALHR	0.52	S protein
HLA-DRB5*01:01	345	359	TRFASVYAWNRKRIS	0.52	S protein
HLA-DRB1*07:01	166	180	KEITVATSRTLSYYK	2.1	M protein
HLA-DRB4*01:01	298	312	YKHWPQIAQFAPSAS	11	N protein

### B cell epitope prediction

For the prediction of B cell epitopes, ABCpred server was used. Based on the binding score (>0.9), non-allergenicity and non-toxicity, a total of four B cell epitopes were finally selected, as shown in **Table 3**.

**Table 3.** Predicted linear B cell epitopes, binding score better than 0.9, are only selected for the final vaccine construct.

Peptide sequence	Predicted score	Polyprotein
AGTITSGWTFGAGAAL	0.97	S protein
GVSVITPGTNTSNQVA	0.95	S protein
TRRIRGGDGKMKDLSP	0.94	N protein
KSAAEASKKPRQKRTA	0.93	N protein

### Construction of multiepitope based vaccine

**Figure 1.**
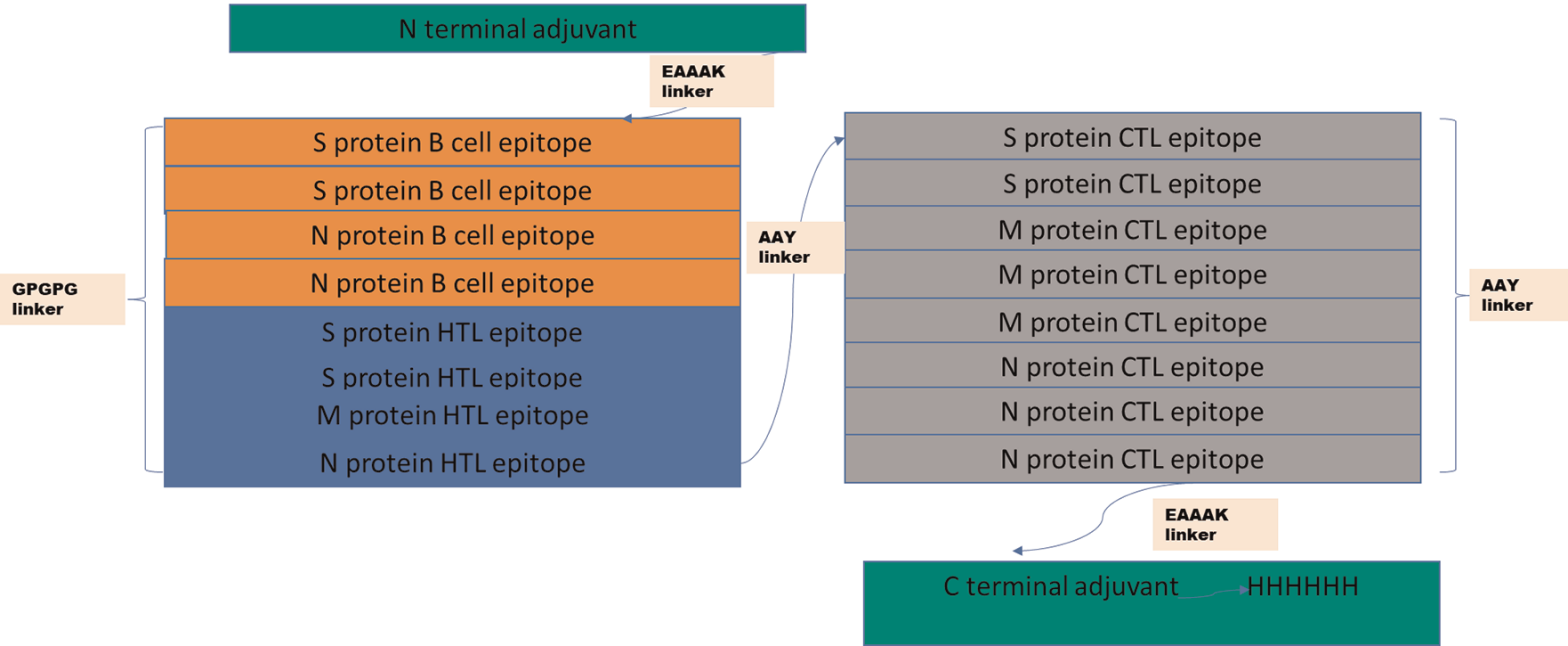
Structure of the final multiepitope based vaccine. At the C terminal, an adjuvant human B defesin3 has been added, and then it is linked with B cell epitopes using EAAAK linkers. B cell epitopes are linked with HTL with GPGPG linkers, and HTL are linked with CTL with AYY linkers. At N terminal, another adjuvant human B defensin-2 has been linked with six histidine sequences.

The four B cell epitopes, four HTL epitopes and 8 CTL epitopes were selected for vaccine construction, which fulfilled all the criteria of binding affinity, antigenicity, non-toxicity and non-allergenicity. Besides these epitopes, two adjuvants were also added at the N terminal (human beta defensin-3) and at C terminal (human beta defensin-2) of the vaccine to increase the antigenicity. Adjuvant were linked via EAAAK linkers to the epitopes, HTL epitopes were linked via GPGPG linkers, while CTL epitopes were linked with AAY linkers, as shown in **Figure 1**. The constructed vaccine sequence was again checked for antigenicity, non-allergenicity, non-toxicity and it fulfilled all the criteria.

### Prediction of physicochemical parameters of the constructed vaccine sequence

The physiochemical parameters of the vaccine sequence were predicted by the ProtParam server. The molecular weight of the construct was predicted to be 38.8 KDa, and the theoretical PI value was 9.92. The predicted half-life in *E. coli* was more than 10 hours, and the instability index in the test tube was found to be stable. The aliphatic value of the vaccine sequence was 58.7 and the grand average of hydropathicity (GRAVY) was -0.348.

### Secondary structure prediction of the vaccine sequence

Secondary structure prediction was made using the CFSSP web tool. The result showed the presence of helix at 44.5%, sheet at 35.6%, and turns at 14 %.

### Tertiary structure prediction of the vaccine sequence

**Figure 2.**
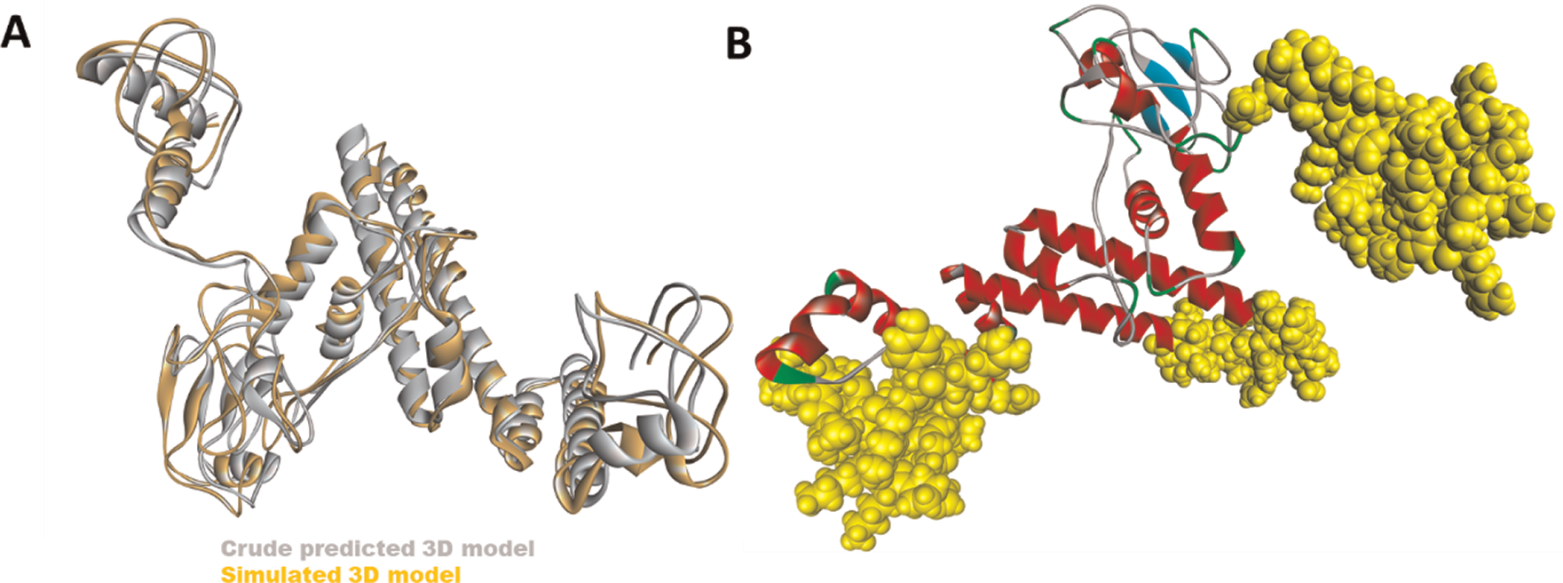
**(A)** The crude 3D modelled structure of the vaccine (grey) has been superimposed with the simulated model (yellow). ( **B)** The top 3 conformational B cell epitopes predicted in the vaccine has been shown with yellow spheres.

**Figure 3.**
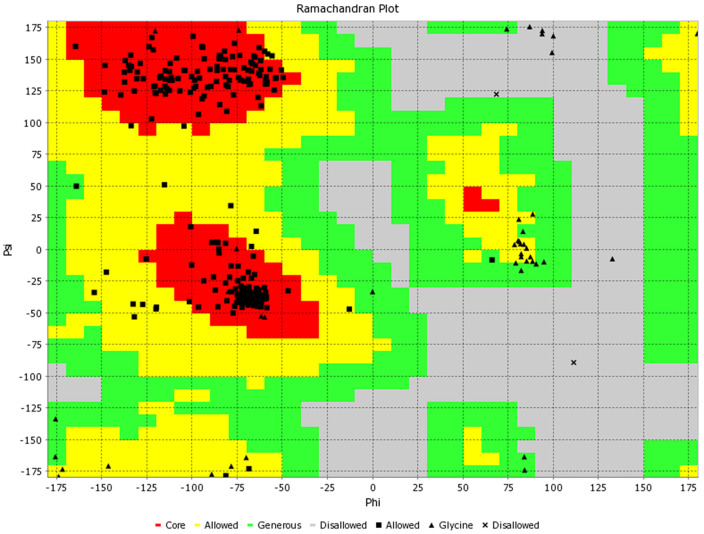
Ramachandran plot of the 3D modelled vaccine construct.

The 3D structure of the multiepitope predicted vaccine was predicted using the Rosetta web tool. It uses de-novo structure prediction using deep neural network algorithm to predict the inter-residue distances as well as orientations. Then these orientations are converted to smooth inter-residue constraints followed by gradient descent energy minimization. Further, coarse-grained models are generated, and full atom refinement is done. It gave five best-predicted models, and based on the TM score, one model was selected for further investigation, as shown in **Figure 2A**. Further to validate the predicted model, Ramachandran plot analysis was done, and results showed that 96.3% residues were in the favourable region, 2.5% were in the allowed region while ~ 1% were in the outlier region ( **Figure 3**). Additionally, the PROSA web tool was used to predict the quality of the modelled vaccine, which predicted a Z score of -6.34. Ramachandran plot and Z score have suggested that the predicted model of protein was valid and could be taken for further analysis.

### Conformational B cell epitope analysis from modelled vaccine

**Figure 4.**
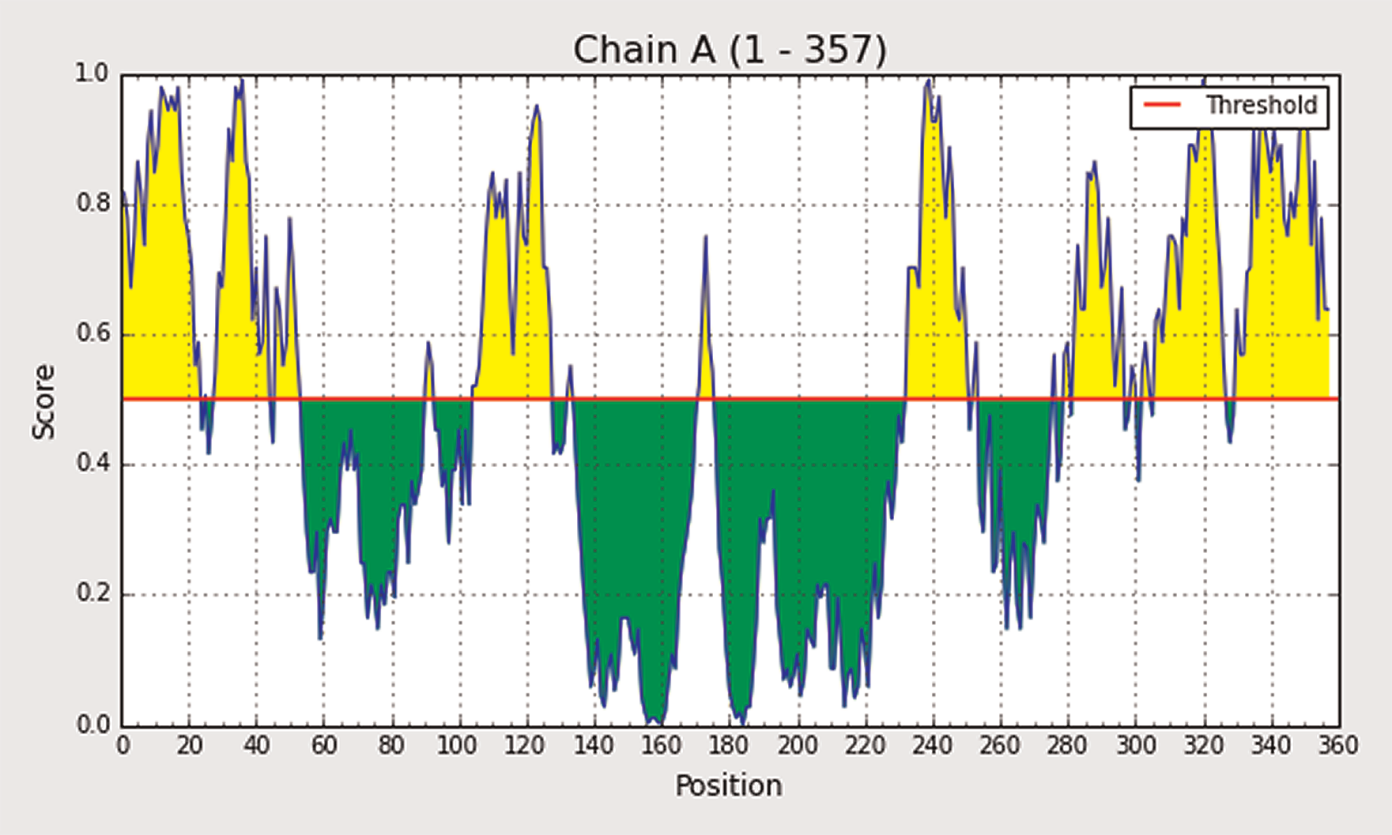
The individual score of discontinuous B cell epitopes predicted in the modelled vaccine

Elipro predicts the antibody epitopes taking protein 3D structure as input. Linear B, as well as discontinuous conformational epitopes, were identified in the vaccine construct using ElliPro, an online server. A total of 8 linear epitopes were predicted, and the sequence of the top 3 epitopes have been reported in **Table 4** and has been shown structurally in **Figure 2B**. Various discontinuous epitope residues were predicted from vaccine sequence length 232-253 (21 epitope residues), between 299-357 (55 epitope residues), between 1-54 (52 epitope residues), between 69-128 (33 epitope residues) and between 168-176 (9 epitope residues) were predicted. The individual score of each of the discontinuous epitopes has been shown in **Figure 4**.

**Table 4.** Predicted top three conformational B cell epitopes from the 3D modelled vaccine construct.

Start	End	Peptide sequence	score
329	357	FCPRRYKQIGTCGLPGTKCCKKPHHHHHH	0.78
232	253	VYAAYAGDSGFAAYAAYLVGLM	0.74
1	54	GIINTLQKYYCRVRGGRCAVLSCLPKEEQIGKCSTRGRKCCRRKKEAAAKAGTI	0.73

### Docking of vaccine with TLR-3 receptor

**Figure 5.**
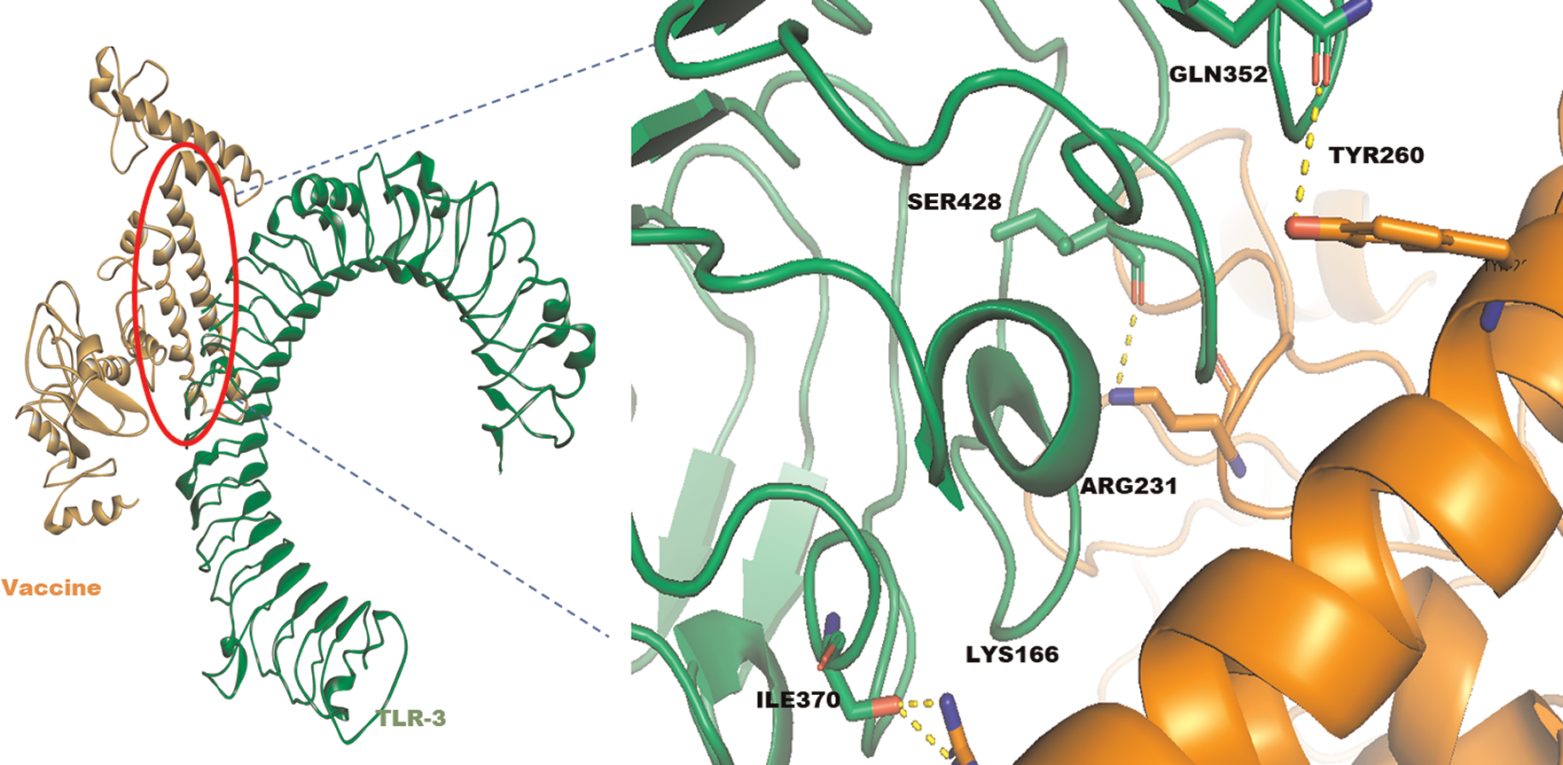
The docked structure of TLR-3 (green)-modelled vaccine (orange) complex illustrating critical residues involved in the interactions.

The modelled structure of the vaccine was taken through energy minimization, equilibration, and MD simulations before docking. The last frame from the simulated trajectory was taken further for docking. The simulated structure has been compared with the crude modelled structure, as shown in **Figure 2A**. The TLR-3 structure was retrieved from PDB (ID 1ZIW). The downloaded structure was prepared and processed for docking using the dock prep tool UCSF Chimera software ^50^. The simulation was done using the PatchDock server and further refined using FireDock. The best-docked complex had global energy of -14.91 Kcal/mol, and attractive Van der Waal’s energy was -18.1 Kcal/mol, which shows a decent binding affinity of the vaccine towards TLR-3. Further, the best binding pose was investigated for polar interactions using discovery studio visualizer ^51^ between TLR-3 and vaccine, and it was found that GLN352, SER428, ILE370 of TLR-3 was making the hydrogen bond with TYR260, ARG321, and LYS166 of vaccine respectively ( **Figure 5**).

### Reverse translation and codon optimization

Jcat was used for the optimization of the codon for the proper expression of the protein. *E. coli* strain K12 was chosen as a host, with additional options such as avoid rho-independent transcription terminators, avoid prokaryotic ribosome binding sites, and avoid Cleavage Sites of Restriction Enzymes. The CAI gives the information of codon usage, generally score between 1 and 0.8, while GC contents should be between 40 % to 70%, values lie outside the given margin is suggested to be inefficient. CAI of the optimized nucleotide sequence of the vaccine was found to be 0.92, with a GC content of 55.6%, which indicates the effective expression of the protein in the *E. coli.*

## Discussion

The structural polyproteins from SARS-CoV-2 were selected for developing a multiepitope based vaccine. Initially, four structural proteins were chosen, namely spike, membrane, envelope, and nucleocapsid protein, based on their antigenicity prediction, role in facilitating the entry of the virus, and packaging inside the host cells. However, when CTL, HTL, and B cell epitopes were predicted, it was found that epitopes from envelope protein were not able to satisfy the criteria of non-allergenicity and antigenicity simultaneously, and hence envelope protein was not considered further in the study. While epitopes selected from SMN structural polyprotein were satisfying, all the criteria such as antigenicity, non-allergenicity, non-toxicity, and high binding affinity towards MHC and also HTL epitopes were able to induce IFN gamma immune response. The constructed multiepitope vaccine from the selected epitopes from all the three SMN polyproteins was again investigated for antigenicity, and it was found that vaccine construct predicted to be a potent antigen with score 0.60 (predicted by Vaxijen). Further, the vaccine construct was looked for its allergenicity, and it was found that the vaccine was a non-allergen with a score of -0.59 (threshold was set on -0.4, predicted by AlgPred tool). Further physicochemical parameters were analysed for vaccine sequence, and it was predicted to have a molecular weight of 38.8 KDa, PI of 9.92, and half-life inside the *E. coli* >10 hours, which shows that protein can easily express and isolated. The 3D model of the constructed vaccine sequence was predicted from the Rosetta web server. Eight conformational linear B cell epitopes were found in the modelled structure of the vaccine, as predicted by the Elipro web tool. It was evident from the prediction that the constructed vaccine model could easily produce adaptive immune response specific to the SARS-CoV-2 antigens. Further, to investigate the ability of the modelled vaccine to interact with TLR receptors on immune cells, the TLR-3 receptor was docked with the modelled vaccine. The results showed that the modelled vaccine had a good binding affinity towards TLR-3, and it was found that GLN352, SER428, ILE370 of TLR-3 was making the hydrogen bond with TYR260, ARG321, and LYS166 of vaccine, respectively. This interaction of vaccine with TLR-3 was predicting that vaccine have the potential to generate both innate as well as adaptive humoral and cell-mediated immune responses. For efficient expression of the protein inside the *E. coli*, codon optimization was done to improve the translation and transcription efficiency. The constructed vaccine sequence was reverse translated, and CAI and GC content were assessed, taking *E. coli* (K12) as a host organism. The CAI index of 0.92 and GC content of 55.6 % and half-life was already predicted to be more than 10 hours, suggests the efficient expression of recombinant protein inside the *E. coli.* This immunoinformatics study suggests that the predicted vaccine could generate specific adaptive immunity against SARS-CoV-2 and could provide a valuable contribution to the management of the COVID-19. This predicted vaccine candidate strongly warrant in-vitro and in-vivo study for the practical implications.

## Conclusions

In this study, a multiepitope (CTL, HTL, and B cell) vaccine construct has been predicted and modelled through immunoinformatics techniques. The predictions suggest that the constructed vaccine could generate both humoral and cell-based adaptive immunity towards SARS-CoV-2. Further, it was also predicted that it may easily be expressed inside the *E. coli* strain (K12). This immunoinformatics study may reduce the expenditure and time for vaccine research and may give a significant value in the management of COVID-19. This in silico prediction warrants the in-vitro and in-vivo study to test the practical implications of the predicted vaccine.

## Data availability

### Underlying data

NCBI: Severe acute respiratory syndrome coronavirus 2 isolate Wuhan-Hu-1, complete genome, Accession number NC045512.2: https://www.ncbi.nlm.nih.gov/nuccore/NC_045512.2/

NCIB: surface glycoprotein [Severe acute respiratory syndrome coronavirus 2], Accession number YP_009724390.1: https://www.ncbi.nlm.nih.gov/protein/YP_009724390.1/

NCBI: membrane glycoprotein [Severe acute respiratory syndrome coronavirus 2], Accession number YP_009724393.1: https://www.ncbi.nlm.nih.gov/protein/YP_009724393.1

NCBI: nucleocapsid phosphoprotein [Severe acute respiratory syndrome coronavirus 2], Accession number YP_009724397.2: https://www.ncbi.nlm.nih.gov/protein/YP_009724397.2

Protein Data Bank: Human Toll-like Receptor 3 extracellular domain structure, Accession number 1ZIW: http://doi.org/10.2210/pdb1ZIW/pdb
