# Supplementary material for: Reverse vaccinology approach towards the in-silico multiepitope vaccine development against SARS-CoV-2
Source: F1000Res. 2021 Jan 23;10:44. [Version 1] doi: 10.12688/f1000research.36371.1 (PMC8009247; doi:10.12688/f1000research.36371.1)
Supplement: Peer review history

# Peer review: Reviewer response for version 1

**DOI:** 10.5256/f1000research.39433.r77969

**Published:** 2021-02-17

**Authors:** Tirumala Bharani K. Settypalli

**Affiliations:** 1 Department of Nuclear Sciences and Applications, Joint FAO/IAEA Division of Nuclear Techniques in Food and Agriculture, International Atomic Energy Agency, Vienna, Austria

The current research article by Kumar *et al.*, utilized *in silico *Reverse vaccinology approach to develop multiepitope vaccine against SARS-CoV-2. Overall, the manuscript was well written and is a valuable contribution at this time of the pandemic.

 Given the pandemic situation, the *in silico *approach is a reliable and fast option for quicker vaccine development. The authors have utilized various immunoinformatic online tools to predict T-cell and B-cell epitopes from three structural polyproteins and constructed vaccine sequence. 

 Although the current study lacks the *in vitro / in vivo* studies, the immunoinformatic approach is helpful to reduce the cost and time for further experimental validation and can be a valuable contribution during the pandemic.

Is the work clearly and accurately presented and does it cite the current literature?

Yes

If applicable, is the statistical analysis and its interpretation appropriate?

Yes

Are all the source data underlying the results available to ensure full reproducibility?

Yes

Is the study design appropriate and is the work technically sound?

Yes

Are the conclusions drawn adequately supported by the results?

Yes

Are sufficient details of methods and analysis provided to allow replication by others?

Yes

Reviewer Expertise:

Molecular genetics, Immunology

I confirm that I have read this submission and believe that I have an appropriate level of expertise to confirm that it is of an acceptable scientific standard.

# Peer review: Reviewer response for version 1

**DOI:** 10.5256/f1000research.39433.r77965

**Published:** 2021-02-09

**Authors:** Sivaraman Natarajan

**Affiliations:** 1 Department of Computational Biology, St. Jude Children’s Research Hospital, Memphis, TN, USA

Kumar et al. in the manuscript attempted in-silico vaccine development against SARS-CoV-2 using publicly available SARS-CoV2-Sequence. They used three different crucial structural proteins (glycoproteins) from Helper T cell, Cytotoxic T cell, and B cell epitope. The epitope was predicted using publicly available tools. After predicting the epitope, they attempted an in-silico vaccine construction by applying various conditions including adding linked adjuvants to predict the efficiency of the vaccine. This immunoinformatics approach is very interesting given the SARS-CoV-2 evolution of new mutant strains, help to predict effective vaccine *in-silico.*

 As the author mentioned in the manuscript it lacks any *invitro* experiment to confirm any of the predictions as proposed in the manuscript. However, the in-silico finding from the study will be immensely beneficial to the researcher who studies the SARS-Co-V2.

Is the work clearly and accurately presented and does it cite the current literature?

Yes

If applicable, is the statistical analysis and its interpretation appropriate?

Not applicable

Are all the source data underlying the results available to ensure full reproducibility?

Yes

Is the study design appropriate and is the work technically sound?

Yes

Are the conclusions drawn adequately supported by the results?

Partly

Are sufficient details of methods and analysis provided to allow replication by others?

Partly

Reviewer Expertise:

Genomics, Single-cell Technology, and Cancer Biology

I confirm that I have read this submission and believe that I have an appropriate level of expertise to confirm that it is of an acceptable scientific standard.
